# Microfluidic-Based Detection of AML-Specific Biomarkers Using the Example of Promyelocyte Leukemia

**DOI:** 10.3390/ijms21238942

**Published:** 2020-11-25

**Authors:** Benedikt Emde, Heike Kreher, Nicole Bäumer, Sebastian Bäumer, Dominique Bouwes, Lara Tickenbrock

**Affiliations:** 1 Department Hamm 1, Hamm-Lippstadt University of Applied Science, 59063 Hamm, Germany; lara.tickenbrock@hshl.de; 2 Micronit GmbH, 44263 Dortmund, Germany; heikekreher@gemail.com (H.K.); dominique.bouwes@micronit.de (D.B.); 3 Department of Medicine A, Hematology and Oncology, University of Muenster, 48149 Muenster, Germany; nbaeumer@uni-muenster.de (N.B.); baumers@uni-muenster.de (S.B.)

**Keywords:** personalized diagnostics and medicine, bio-microfluidics, lab-on-a-chip, acute myeloid leukemia, magnetic bead-based immunoassay

## Abstract

A microfluidic assay for the detection of promyelocytic leukemia (PML)-retinoic acid receptor α (RARα) fusion protein was developed. This microfluidic-based system can be used for rapid personalized differential diagnosis of acute promyelocyte leukemia (APL) with the aim of early initiation of individualized therapy. The fusion protein PML-RARα occurs in 95% of acute promyelocytic leukemia cases and is considered as diagnostically relevant. The fusion protein is formed as a result of translocation t(15,17) and is detected in the laboratory by fluorescence in situ hybridization (FISH) or reverse transcriptase polymerase chain reaction (RT-PCR). Diagnostic methods require many laboratory steps with specialized staff. The developed microfluidic assay includes a sandwich enzyme-linked immunosorbent assay (ELISA) system for PML-RARα on surface of magnetic microparticles in a microfluidic chip. A rapid detection of PML-RARα in cell lysates is achieved in less than one hour. A biotinylated PML-antibody on the surface of magnetic streptavidin coated microparticles is used as capture antibody. The bound translocation product is detected by a RARα antibody conjugated with horseradish peroxidase and the substrate QuantaRed. The analysis is performed in microfluidic channels which involves automated liquid processing with stringent washing and short incubation times. The results of the developed assay show that cell lysates of PML-RARα-positive cells (NB-4) can be clearly distinguished from PML-RARα-negative cells (HL-60, MV4-11).

## 1. Introduction

In microfluidics, liquids are processed in structures in the submillimeter range [1,2]. The behavior of liquids in microfluidics differs fundamentally from the behavior in the macroscopic range, because frictional forces dominate inertial forces, resulting in laminar flows and avoiding turbulent flows [2]. In laminar flows, only a diffusion-driven mixing of fluids takes place [2]. The capillary forces also dominate over the weight force. Small quantities of liquids can thus be moved against the force of gravity [2]. This technology is particularly used in diagnostics and biological research [3]. Thus, miniaturization can increase the performance of biological assays. An increased surface-to-volume ratio increases molecular diffusion and heat transfer, whereas the molecular reaction remains unchanged [4]. This results in faster and more sensitive diagnostics [4].

Microfluidic systems that combine several laboratory steps on a microfluidic chip are called lab-on-a-chip systems or micro total analysis system (µTAS systems). Several functionalities, for example the microfluidic chip and non-fluidic parts, are combined [4]. Lab-on-a-chip systems, which are intended for direct use on patients, are called point-of-care systems (POC). The lab-on-a-chip system must function without infrastructure as well as independent of location and without the typical laboratory [5]. Lab-on-a-chip and point-of-care systems are characterized by their short analysis times, automated process steps, and low sample and reagent volumes [4,6]. The short analysis times mean that a tailor-made therapy can be carried out more quickly [5]. This has a direct influence on the patients’ chances of survival [5,7]. The low use of assay reagents and sample material does not only save money, but also avoids cost-intensive techniques for the collection of patient samples. The automated processing of liquids in the lab-on-a-chip system ensures user-independent analysis and also prevents contact with hazardous chemicals or infectious patient material.

Acute promyelocytic leukemia (APL) is a subtype of acute myeloid leukemia (AML) [8]. APL is often characterized by a chromosomal translocation of the chromosomes 15 and 17 t(15; 17) [8,9,10]. This concerns the genes retinoic acid receptor α (RARα) and promyelocytic leukemia (PML) and results in the formation of the translocation product PML-RARα [8,10]. This subtype of AML is also known as French–American–British classification system (FAB) type M3 [11]. Translocation t(15;17) occurs in approximately 95% of APL patients. Other PML-RARα fusion genes occur due to chromosomal aberrations [11,12]. Patients with APL often have pronounced coagulation disorders, which are associated with a high risk of life-threatening intracerebral bleeding and bleeding in the skin and mucous membranes, gastrointestinal tract and lungs [13,14]. Classic external characteristics are fatigue, loss of performance and paleness. The APL is considered AML which is particularly aggressive and life-threatening [13,14].

Simultaneous neutropenia also leads to an increased tendency to infection. Differential diagnosis is performed when APL is detected by reverse transcriptase polymerase chain reaction (RT-PCR) or fluorescence in situ hybridization (FISH) [8,15,16]. The fusion gene PML-RARα is detected.

In addition to the possibility of the detection of the translocation on DNA level via the fusion gene, it is also possible to detect the translocation product PML-RARα on protein level. The method of a sandwich enzyme-linked immunosorbent assay (ELISA) is suitable for this purpose. In a sandwich ELISA a capture antibody on the surface of a matrix is used to bind the analyte specifically [17]. A second antibody labelled with an enzyme which is specific for another epitope of the analyte is added and binds to the antibody–antigen complex [17]. The two antibodies form the name-giving sandwich. The result of the binding of the analyte to the antibodies can now be quantified with a substrate for the enzyme on the detection antibody [17]. This method is highly specific, because only a positive signal is obtained when both antibodies have specifically bound the antigen to different epitopes [17,18]. Non-specific binding is reduced by an optimized number of washing steps. A sandwich ELISA can be used to detect a target protein in unpurified samples with antibodies against different epitopes [17].

Another method is the immunobead assay. The beads are coated with a specific antibody against the target protein. The bead-antibody complex binds the protein. Another antibody, labeled with a fluorescent dye specific against the bounded protein, is added for detection [19,20]. The detection of the fluorescence intensity takes place in a flow cytometer [19,20]. A sandwich procedure is also used in this assay. The difference to the sandwich ELISA is the use of a fluorescent dye instead of an enzyme to detect antigen binding. With this technique, assays for the diagnosis of the fusion proteins BCR-ABL and PML-RARα have already been developed [19,20].

APL can also be diagnosed by staining of PML nuclear bodies. The PML protein is involved in the regulation of cell growth and apoptosis [21]. PML forms PML nuclear bodies in the nucleus of most cells [21]. The nuclei of eukaryotic cells contain, on average, 5–30 PML nuclear bodies [22]. PML nuclear bodies can be stained with an anti-PML antibody labeled with a dye. In APL, the formation of PML nuclear bodies is disturbed. This changes the nuclear staining pattern [23] and APL can be diagnosed. An analysis with this method takes about 4 h. Dimov et al. have tested this detection method in a study over 12 years on 349 cases [24].

Patients with APL are treated with a combined therapy of all-trans-retinoic acid (ATRA) and arsenic trioxide (ATO). ATRA leads to a differentiation of malign cells and is a non-cytotoxic drug. The therapy of APL with ATRA is considered the first targeted therapy and is an example of personalized medicine [10]. This therapy has made it possible to achieve a long-term survival rate of over 90% for patients with a newly diagnosed APL [10,25].

The diagnosis of APL is of utmost importance for effective patient care by early initiation of a tailor-made therapy. Due to the acute and life-threatening bleeding, a diagnosis of APL should be made in a few hours and not in several days. Cytomorphological examinations, immunophenotyping combined with karyotyping are usually accomplished within one day. Molecular genetic analysis using RT-PCR and FISH usually take 1–3 days, as these are limited to specialized laboratories and well qualified staff. Not every laboratory is able to perform these analyses. Due to these points, new specific detection methods for PML-RARα are necessary. We have developed a method to detect the fusion protein PML-RARα in cell lysates by a sandwich ELISA on the surface of magnetic particles in a microfluidic chip in less than one hour.

## 2. Results

### 2.1. Magnetic Bead-Based Sandwich ELISA

**Figure 1:**
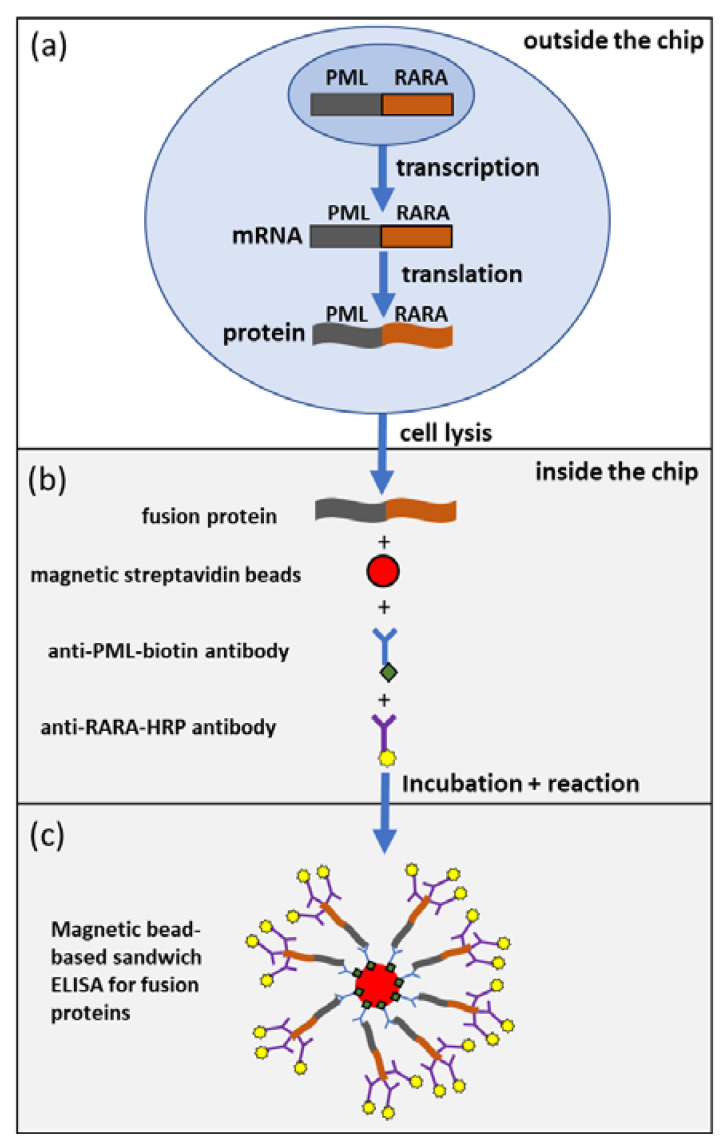
Schematic representation of the bead-based sandwich enzyme-linked immunosorbent assay (ELISA) for promyelocytic leukemia (PML)-retinoic acid receptor α (RARα). First the expression of PML-RARα in NB-4 cells is shown with subsequent cell lysis outside microfluidics (**a**). The cell lysate is then incubated with magnetic streptavidin beads, anti-PML-biotin antibodies and anti-RARα- horseradish peroxidase (HRP) antibodies in the microfluidic chip (**b**). The anti-PML-biotin antibodies bind to the magnetic streptavidin beads. The anti-PML antibody binds to the PML region of the fusion protein while the anti-RARα-HRP antibody binds to the RARα region (**c**). After different washing steps, the reduction of resazurine to resorufine catalyzed by HRP leads to the detection of fluorescence intensity in case of the presence of the fusion protein.

The developed bead-based sandwich ELISA uses anti-PML and anti-RARα antibodies against regions of PML-RARα which occur in all forms of PML-RARα fusion proteins. Anti-PML was used as capture antibody on the surface of magnetic particles and RARα as detection antibody coupled with horseradish peroxidase (HRP). The prototype of the bead-based sandwich ELISAS is shown schematically in Figure 1. Both anti-PML and anti-RARα antibodies were biotinylated and modified with HRP and used as capture antibodies on the surface of magnetic particles or detection antibodies. The best results were obtained with the anti-PML-biotin antibody as capture antibody and the anti-RARα-HRP antibody as detection antibody. This setup was used in the assay and the subsequent results were obtained.

The assay for the detection of PML-RARα was established in reaction tubes, multiwell plates and in a microfluidic chip. The specificity of the assay was determined by the detection of PML-RARα in t(15;17)-positive leukemic cell lines. Buffers and other t(15;17)-negative leukemic cell lines served as negative controls. Only the t(15;17)-positive cell line showed an increased fluorescence intensity at an excitation of 570 nm and an emission of 585 nm. To prove the principle of the assay, shown in Figure 1, 10 µg–0.1 µg total cell lysate was used per test batch. This corresponds to a concentration of 50 ng/µL–0.5 ng/µL of the total cell lysate per test batch.

### 2.2. Microfluidic Chip, Chip Holder, and Liquid-Handling-System

**Figure 2:**
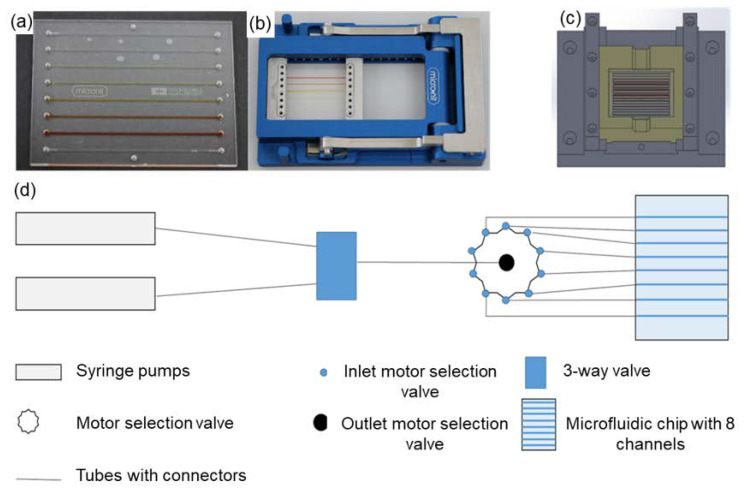
Illustration of the microfluidic chip with chip holder and chip slider as well as schematic representation of the liquid handling system. The microfluidic chip, developed by Micronit GmbH (Dortmund, Germany), consisting of 8 channels with inlets and outlets, is shown in part (**a**). In (**b**), the microfluidic chip can be seen in the individually adapted chip holder. By inserting tubes with 1/16” outer diameter into the connectors, it is possible to fill the channels without mixing via sealing rings. The magnetic slider is shown at (**c**). The chip carrier is shown brightly and can be moved over the embedded magnet so that each channel is aligned parallel to the magnet. The liquid handling system is shown schematically under (**d**). The syringe pumps are connected to the 3-way valve so that both liquids can continue into the motor selection valve. By switching the motor selection valve, the inlet can be connected to any outlet of the valve. The pumps and valves are controlled by a laptop using LabView. This liquid handling system allows liquids from the syringe pumps to be applied to each microfluidic channel at a defined flow rate and quantity.

In order to create the biological system (Figure 1), we tried to develop the right microfluidic chips (Figure 2). The microfluidic chip measures 50 mm × 35 mm with eight microfluidic channels integrated at a pitch of 2 mm. The channels are filled via inlets on the upper side (Figure 2a). The chip holder is used to connect the tubes with the microfluidic chip. Tubes with an outside diameter of 1/16′ are pushed into a connector with eight holes and sealing rings. The chip is clamped under the connector and sealed tightly (Figure 2b) by light lifting action.

The liquid handling system is used to manipulate the fluids in the channels and fill the channels with the required assay fluids (Figure 2d). The flow rate and pump duration can be adjusted at the syringe pumps. The quantity to be applied is defined via a sample loop in the three-way valve. All channels can be controlled individually via a motor selection valve. Both the pumps and the valves are connected to a laptop via a Recommended Standard 232 (RS232) interface and controlled via LabView.

### 2.3. Magnetic Bead Manipulation

In the assay in reaction tubes, the magnetic particles are manipulated via a magnetic stand. The reaction tubes are manually placed in the stand at each washing step, the assay components removed with a pipette and the beads resuspended with wash buffer. In the multiwell plate format, the entire plate is placed on another plate with magnets so that magnets are located under the cavities to be processed. The solutions are removed and added by pipetting. A magnetic slider was also developed for the microfluidic chip (Figure 2c). This contains a 40 mm-long neodymium magnet which is positioned parallel to the channels on the microfluidic chip. The chip is placed in a movable carriage above the magnets. The carriage is pushed into a dovetail groove and can be locked by positioning holes orthogonal to the channels so that each channel can be aligned parallel to the magnet.

**Figure 3:**
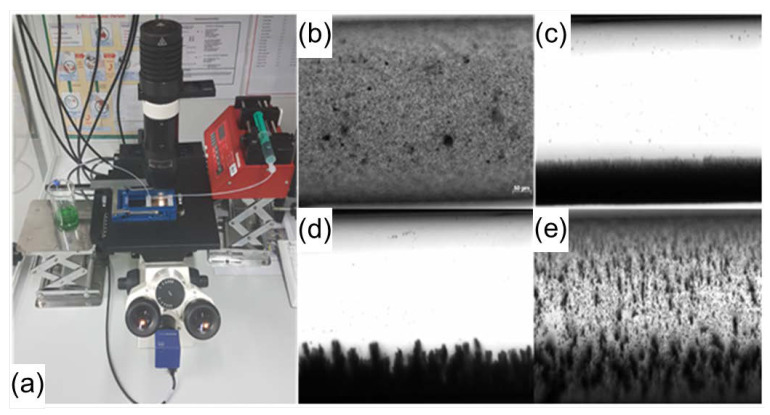
Fluidic analysis of magnetic beads through the microfluidic chip. Shown is the analysis of the fluidic behavior of the magnetic beads in the microfluidic channel and the influence of a neodymium magnet on them. Under (**a**) the experimental setup consisting of a syringe pump, tubes, connectors and a microfluidic chip in the chip holder is shown. The following pictures were taken with the microscope axiovert 40 CFL. The first image (**b**) shows the magnetic beads evenly spread in the channel at 100× magnification. The syringe pump is switched off and there is no flow rate. The next microscopic image (**c**) shows the beads in the channel with a magnet parallel to the channel. The volume flow is 10 µL/min. The magnetic beads form an approx. 80 µm-wide layer along the channel (black arrow). Due to the magnetic forces, this layer adheres to the channel wall and the beads are not flushed out of the channel. If the magnet is positioned about 5 cm away from the channel, the attraction to the beads is reduced. The bead layer parallel to the channel is no longer as homogeneous and dense (grey arrow, (**d**)). The flow rate is again 10 µL/min. Beads that reach far into the channel are rinsed out of the channel by the fluid flow. In the last part of figure (**e**), the pump was switched off and the magnet was removed. The beads begin to spread evenly in the channel again. The process shown in Figure 1 can be used to implement washing steps for magnetic beads in the microfluidic channel.

The magnetic manipulation of the magnetic beads is shown in Figure 3. The behavior of the beads is documented under a microscope at 100× magnification (Figure 3a). The beads are injected into the channel and spread evenly (Figure 3b). In Figure 3c, the magnet is aligned under the fluidic chip, parallel to the microfluidic channels. There is a flow rate of 10 µL/min. Figure 3c shows an approximately 80 µm-wide layer of magnetic particles on the channel wall. The particles are not flushed out of the channel. The magnet is then moved 5 cm away from the channel. The bead layer on the channel wall is less homogeneous and dense. At a flow rate of 10 µL/min, particles that reach more than 80 µm into the channel are flushed out of it (Figure 3d). In Figure 3e, the pump is switched of and there is no fluid flow. The magnet is also removed. The beads are now evenly distributed in the channel. This process is used for washing step in the bead-based ELISA for PML-RARα.

### 2.4. ELISA Analysis for PML-RARα in Leukemic Cell Lines

**Figure 4:**
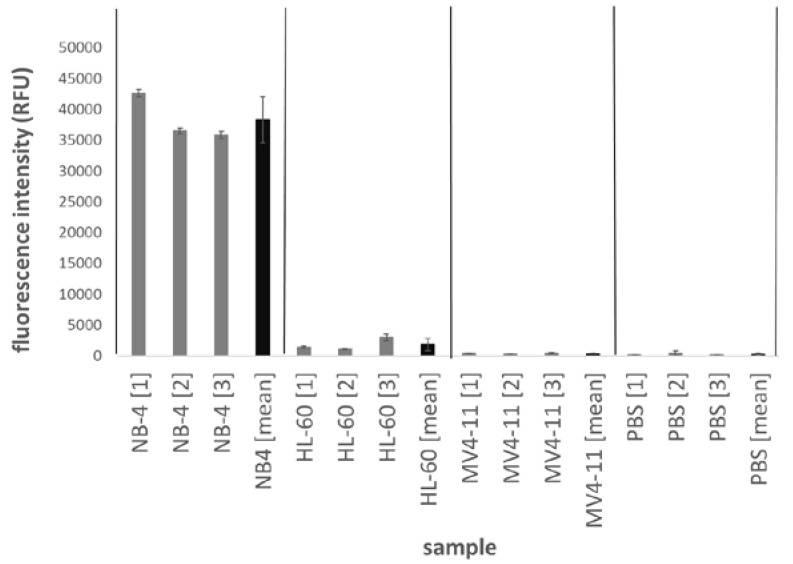
Magnetic bead-based sandwich ELISA for PML-RARα in a reaction tube. Illustrates the results for the sandwich ELISA of PML-RARα on the surface of magnetic beads. The experiments are carried out in reaction tubes. The fluorescence intensity of the test samples is measured in a microtiter plate. From each sample (NB-4, HL-60, MV4-11, PBS) 3 different lysates were measured in triplicates.

**Figure 5:**
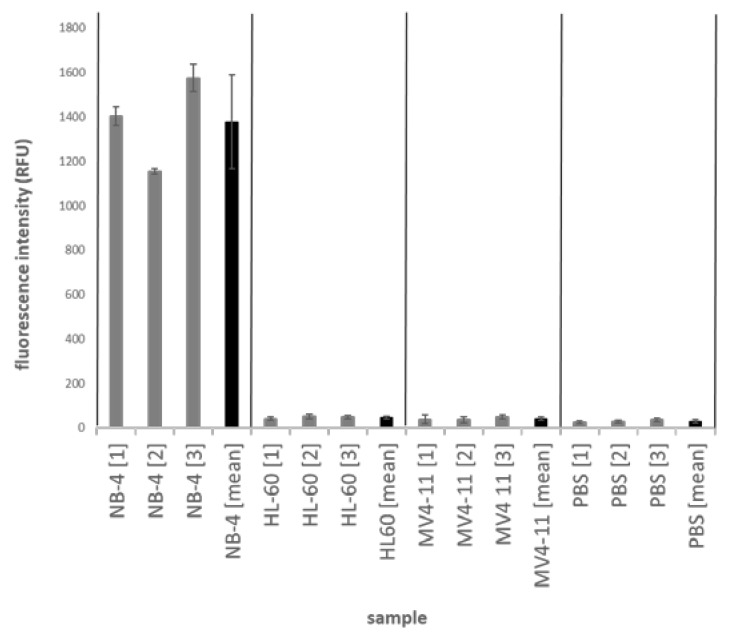
Magnetic bead-based sandwich ELISA for PML-RARα in the microfluidic chip. Shows the results of the sandwich ELISA on magnetic beads in microfluidic channels. Cell lysates of NB-4, HL-60 and MV4-11 were used. In addition, a control with PBS buffer was performed and substrate was measured as an empty value. From each cell line, three different cell lysates were measured in triplicates. The bars represent the standard deviations of the triplicate measurements, which is highest at NB-4 (3) with a value of 60 RFU.

The magnetic bead-based sandwich ELISA was tested with NB-4, HL-60 and MV4-11 cell lines as well as phosphate buffered saline (PBS) buffers as negative control. The assay was performed in reaction tubes (Figure 4) and in microfluidic chips (Figure 5). Three different cell lysates were measured in triplicates. The detection of the reaction tube-based assay was measured in a multiwell plate. The standard deviations for the triplicate measurements as well as for the measurement of the three different lysates were calculated and displayed in the figures. The results in the reaction tube show fluorescence intensity values of 42,500–35,000 relative fluorescence units (RFU) for NB-4 samples with a maximum standard deviation for the triplicates of approx. 600 and between the different NB-4 lysates of approx. 3700.

The negative lysates HL-60 (1850 RFU), MV4-11 (400 RFU) and PBS (170 RFU) have significantly less fluorescence intensity values than the NB-4 cell lysates (Figure 4). The fluorescence intensities of the positive signals in the microchip system are approx. 25 times lower at approx.1400 RFU (Figure 5).

The standard deviation between the results with different positive lysates is about 15% (211 RFU). For HL-60, the mean fluorescence intensity is 47 RFU, for MV4-11 40 RFU and 28 RFU for PBS. The microfluidic chip format also provides a significantly higher fluorescence intensity signal for the positive lysates (NB-4) than it does for the negative controls.

In order to determine whether the higher fluorescence intensity signal depends on the presence of PML-RARα in the positive control, the bead-based sandwich ELISA was performed with a dilution series of NB-4 lysates both in the reaction tubes and in the microfluidic chip under the same conditions. The sensitivity of the assay was also determined by the dilution series of NB-4 lysate t(15;17).

**Figure 6:**
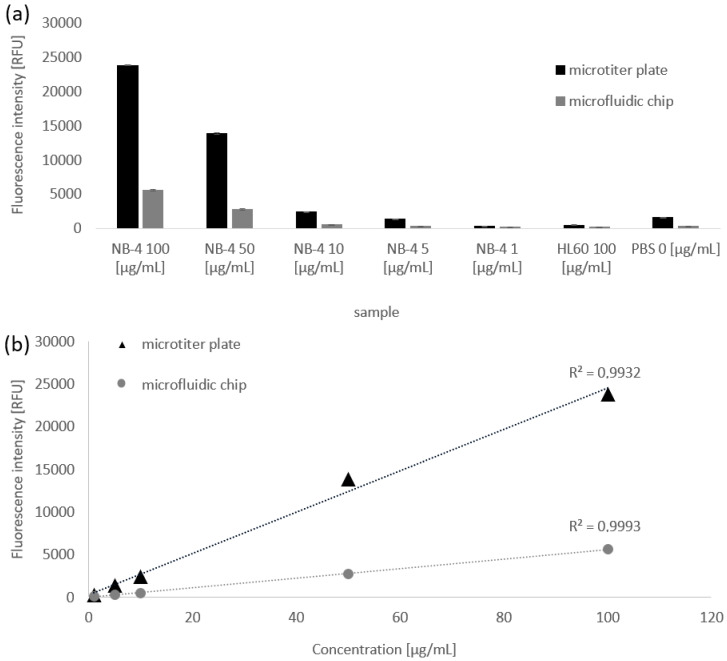
Comparison of magnetic bead-based sandwich ELISAs in reaction tubes and the microfluidic chip for the same sample. The reaction was performed in reaction tubes and then the fluorescence intensity was measured in the multi well plate and the microfluidic channels. Five dilutions of an NB-4 cell lysate in concentrations of 100, 50, 10, 5, 1 µg/mL were used. HL-60 cell lysates were used in a concentration of 100 µg/mL as a negative control. The upper (**a**) part of the figure shows the fluorescence intensities for each sample. The black bars show the intensities in the microtiter plate and the grey bars the intensities in the microfluidic chip. The measured signals are about 5 times higher in the microtiter plate than in the microfluidic chip. The lower part (**b**) of the figure shows an XY-point representation of the individual intensities for the different concentrations in the microtiter plate (black, triangle) and in the microfluidic chip (grey, circle). A regression grade illustrates the linear relationship between the concentration and fluorescence intensity values.

The assay solution in the reaction tube and the microfluidic chip contain 10–0.1 µg total cell lysate. The experiments show that comparable results can be achieved either with the measurement methods in the multiwell plate and the microfluidic chip, although the fluorescence intensity in the microfluidic chip is lower due to the smaller measurement volume (Figure 6a). The concentration measurements were compared to those shown in an XY-point plot (Figure 6b). The regression coefficient is 0.9932 for multiwell plate measurements and 0.9993 for microfluidic channel measurements. This presents the linear relationship between the measurement signal and the concentration of NB-4 lysate in the assay wells. Therefore, it can be assumed that the fluorescence signal is dependent on the NB-4 lysate concentration and thus on the PML-RARα concentration in cell lysates. The results also show that PML-RARα can be detected in total NB-4 cell lysate with a concentration of 5 ng/µL in reaction tube and microfluidic chips. The detection method is also valid for the detection by microfluidic chip.

### 2.5. ELISA Analysis of PML-RARα in Patient Samples

**Figure 7:**
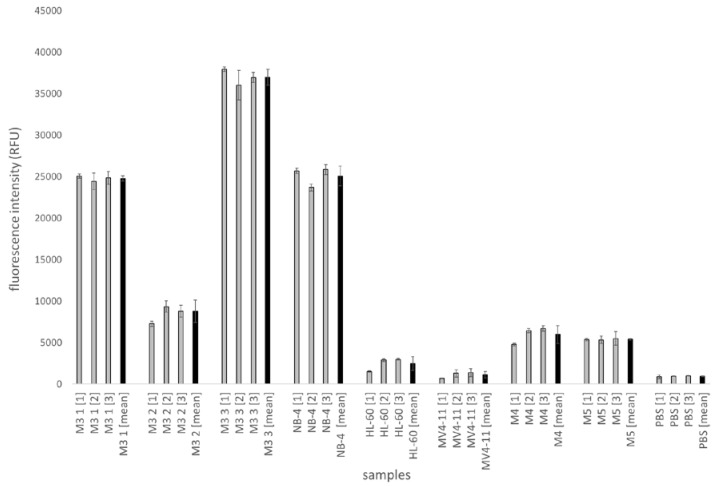
Analysis of patient samples with the magnetic bead-based sandwich ELISA for PML-RARα in multiwell plates. Figure 7 illustrates the results for sandwich ELISA experiments for PML-RARα with magnetic beads in multiwell plates. Fluorescence intensity was measured by a multiwell plate reader. Each sample (M3 (1), M3 (2), M3 (3), M4 (1), M5 (1) as well as control cell lines NB-4, HL-60, MV4-11, PBS) were measured in triplicates.

After testing magnetic bead-based sandwich ELISA with cell lines NB-4, HL-60 and MV4-11, the assay was performed with five primary patient samples. Three FAB type M3 samples and one FAB type M4 and one M5 (PML-RARα-negative samples) were analyzed. The experimental settings and measurements of fluorescence intensities were performed in multiwell plates in triplicates with a total protein concentration of 10 µg/mL each. Standard deviations for the triplicate were calculated and displayed in Figure 7. In addition, mean values were calculated for three triplicate measurements and standard deviation were given. The results in the multiwell plate show fluorescence intensity values of approx. 24,800 RFU for M3 1, 8800 RFU for M3 2 and 37,000 for M3 3 with a standard deviation for the triplicates of approx. a maximum of 1000 (RFU) for M3 1 [2], 700 (RFU) for M3 2 [3] and 1760 (RFU) for M3 3 [2].

Patient samples M4 (1) (approx. 6000 RFU) and M5 (1) (approx. 5300 RFU) have lower fluorescence intensity values than M3 patient samples (Figure 7). The maximum standard deviation of the negative patient samples is approx. 500 RFU. NB-4 (approx. 25,000 RFU), HL-60 (2500 RFU), MV4-11 (1100 RFU) and PBS (900 RFU) were also analyzed as control samples. The results show that the M3 (2) RFU values are significantly lower than the M3 1 and M3 3 and the NB-4 RFU values. It is also notable that values for M4 (1) and M5 (1) are on average approx. two times higher than those of negative controls HL-60, MV4-11 and PBS. However, the values for M4 1 and M5 1 are approximately four times lower than the RFU values of NB-4-positive control. Three independent measurements in triplicates show that the fluorescence intensity values for the samples could be reproduced.

**Figure 8:**
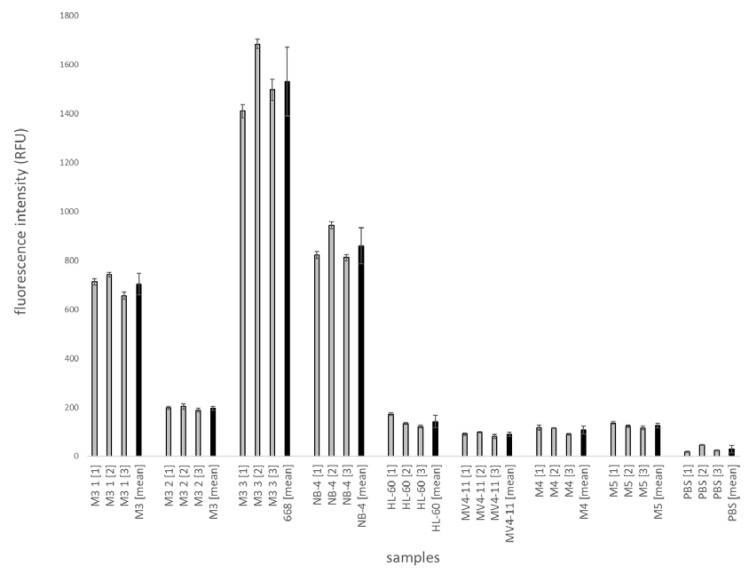
Analysis of patient samples with magnetic bead-based sandwich ELISA for PML-RARα in a microfluidic chip. The analysis was performed in microfluidic chips. Three different M3 lysates and one M4 and M5 lysate were measured as patient samples. Cell cultures NB-4, HL-60 and MV4-11 served as controls. Fluorescence intensities were measured by a multiwell plate reader. Three independent experiments were conducted. Mean values of three experiments were formed from ten measuring points along the microfluidic channel. Black bars show mean values of three experiments. In each case the standard deviation is given.

The same samples were measured in the microfluidic chip (Figure 8). Each sample was used at a concentration of 100 µg/mL. Based on the volume of a reaction mixture, this results in an amount of approx. 0.1 µg lysate per microfluidic channel. Three independent experiments were performed in the microfluidic chip; grey bars show fluorescence intensity values for three independent experiments. Standard deviations of measurements along 10 points of the microfluidic channel were given. Black bars show mean values of three experiments with standard deviation. The means of the analysis for PML-RARα-positive lysates M3 1 show RFU values of about 700 for M3 2 about 200 and for M3 3 about 1530. For the negative lysates M4 and M5 fluorescence, intensities of 110 and 125 RFU were measured. The lysates NB-4 (positive), HL-60 and MV4-11 (both negative) were measured as controls. NB-4 shows a value of 860 RFU, HL-60 of 140 RFU and MV4-11 of 90 RFU. In percentage terms, the standard deviations of all samples exceed 10%.

## 3. Discussion

The presence of the fusion protein PML-RARα is diagnostic evidence for APL. In about 95% of APL patients, the translocation (15;17) is present. APL is a serious disease that can lead to fatal internal bleeding because of coagulation disorder. Rapid diagnosis is necessary so that patients can be treated quickly. This leads to a decrease in mortality.

**Figure 9:**
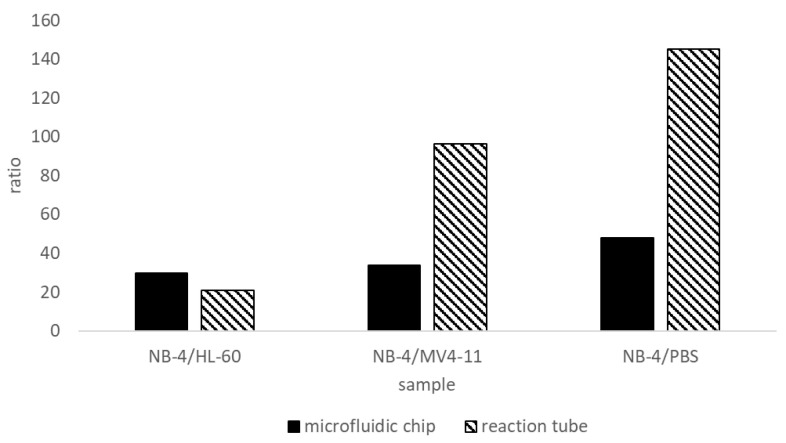
Ratio of the positive to the negative signals of the bead-based sandwich ELISA. Shown is the ratio of positive to negative signals from the magnetic bead-based sandwich ELISA (Results Figure 4 and Figure 5). The lowest positive to negative ratio is 20-fold in the bead ELISA in the reaction tubes, whereas the lowest ratio in the microfluidic chip is 10 times higher at 30-fold (NB-4/HL-60). Since the positive signals are at least 20 times higher than the negative controls, we can speak of specific binding of the fusion protein. The assay is specific for the fusion protein PML-RARα.

We have developed a detection for the fusion protein PML-RARα. PML-RARα is detected by a sandwich ELISA on magnetic particles in the microfluidic chip. The sandwich method increases the specificity of the detection. A specific PML antibody binds at the PML region of the fusion protein and a RARα antibody at the RARα region. The PML antibody is used as capture antibody and the RARα antibody as detection antibody. For a positive signal, a binding of both antibodies is necessary. The cell lysates of PML-RARα-negative cells are positive for the proteins PML and RARα, therefore the sandwich procedure is necessary to verify the presence of the fusion protein. If only PML antibodies were used in the assay, a positive signal would be generated for the wild-type protein PML and the fusion protein PML-RARα. The same problem for the assay exists by using only the RARα antibody. The assay is only specific for PML-RARα by using both antibodies, anti-PML and anti-RARα, in a sandwich system. Western blot analyses of cell lysates of the PML-RARα-positive cell line have confirmed this [26]. The RARα antibody detects PML-RARα and wild-typ RARα in Western blots [26]. In order to assess whether a sample is positive or negative for the fusion protein PML-RARα, the ratio between positive and negative signal is calculated. We have assumed that a positive signal is present if the ratio is at least five. The ratios of the signals are shown in Figure 9 and were generated from the results of Figure 4 and Figure 5.

The ratio of the fluorescence intensities shows that the positive signals are at least 20 times higher than the negative signals. NB-4/HL-60 in the reactions tube shows the worst ratio with 20. This can be explained by unspecific binding of the assay antibodies to the reaction tubes or other assay components. More stringent washing steps can prevent unspecific binding. In the microfluidic chip, washing steps can be performed more stringently and automatically. The ratio for NB-4/HL-60 is 30, approx. 33% higher in the microfluidic chip. The differences between the different cell lysates HL-60 and MV4-11 can be explained by the protein composition of the lysates because the proteins PML and RARα also occur in these cell lysates. The sensitivity of the sandwich ELISA for PML-RARα was determined in relation to the total protein concentration of the cell lysates. This is because the amount of PML-RARα in the cell lysates is unknown. Highly purified fusion protein PML-RARα was not available for development. Unfortunately, there is no commercially available product on the market for the detection of the fusion protein PML-RARα, which could be used as a reference method for comparison. For future analyses, a comparison to DNA based diagnostics like RT-PCR and FISH will be made.

Three samples, which were characterized as FAB M3, were used next to one FAB M4 and one FAB M5 sample. PML-RARα should be present in the FAB M3 samples and a positive fluorescence signal is expected. Samples declared as M4 and M5 according to FAB do not contain PML-RARα. Therefore, it should not show a fluorescence signal that can be evaluated as positive detection.

In order to compare the fluorescence intensities of the patient samples with the fluorescence intensities of the cell line experiments, the cell lines lysates for NB-4 (positive), HL-60 (negative) and MV4-11 (negative) were also analyzed in the same approach. The results show that the fluorescence intensities of M3 (1) and M3 (3) are about the same as those of the positive control NB-4. An exception is the patient sample M3 (2). The fluorescence intensity for M3 (2) is about 8800 RFU in the multiwell plate und 200 RFU in the microfluidic chip, which is about 3 to 4 times lower than the RFU value for NB-4 (about 25,000 and 860). The three to four times lower value of M3 (2) could be explained by a lower concentration of PML-RARα in the protein mixture of the lysate. It is possible that the concentration of M3 blasts in the bone marrow sample of this patient is much lower than in the patients of the samples M3 (1) and M3 (3). As a consequence, the amount of the fusion protein PML-RARα would also be lower and thus lowering the maximum fluorescence intensity to be measured.

It is possible that sample M3 (2) has a different subtype of PML-RARα and the developed bead-based sandwich ELISA is not sensitive to this subtype of fusion protein. Three common isoforms are distinguished, which differ in their breakpoint clusters in three different genomic regions [19]. In the RARα gene, all isoforms have the same break point, which is located in intron 2 [19]. The isoforms therefore differ in the break point cluster region on the PML gene. The isoforms are usually referred to as bcr1 with the break point in intron 6, bcr2 with the breakpoint in exon 6 and bcr3 with the breakpoint in intron 3 [19]. Isoforms of bcr1 occur with a frequency of 55 % and bcr2 with 5 % and bcr3 with 40% [19]. Different isoforms of the fusion genes form several fusion proteins, which can be detected with different sensitivities. NB-4 cells have the most common isoform bcr 1. The functionality and the sensitivity, respectively, of the assay for different subtypes of the fusion protein have to be proven in further studies.

Negative patient samples M4 (1) and M5 (1) show a four to eight times lower signal than the positive control NB-4. Comparing the negative patient samples M4 (1) and M5 (1) with the negative cell lines, it is noticeable that the fluorescence intensity of the patient samples is a maximum of two times higher.

Absolute fluorescence intensity values for analyses in the microfluidic chip are much lower than the values for analyses in the multiwell plate. The fluorescence intensity of the positive control (NB-4) is about 30 times lower than the values in the microfluidic plate. The ratios between the intensity values of the different samples are similar. The lower fluorescence intensity can be explained by the lower measurement volume in microfluidic chip

**Figure 10:**
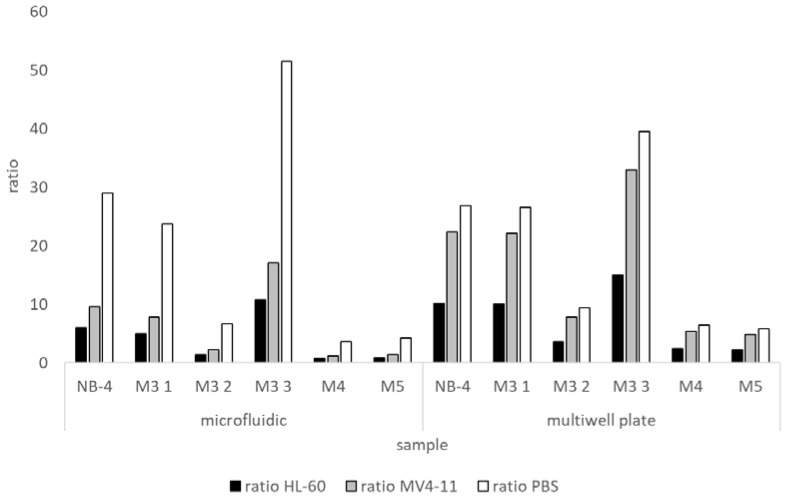
Ratios of fluorescence intensities values of patient samples to negative controls created with the bead-based sandwich ELISA in the multiwell plate (**right**) and microfluidic chip (**left**). Ratios were calculated from the values in Figure 7 and Figure 8 and show ratios of patient samples to the negative controls (HL-60, MV4-11 and PBS). NB-4 was used as a positive control. Ratios for HL-60 are shown in black, for MV4-11 in grey and for PBS in white bars.

For a better interpretation of fluorescence intensity values of the patients’ samples, the ratios of sample to negative controls (HL-60, MV4-11 and PBS) were calculated. Results are shown for the multiwell plate and the microfluidic chip in Figure 10. The figure was created with the data from Figure 7 and Figure 8. Positive/negative ratios show a similar tendency in both the microfluidic chip and the multiwell plate. The positive/negative ratio is lower for HL-60 than for MV4-11 and lower for MV4-11 than for PBS. Looking at the samples, it is remarkable that the samples M3 (1) and M3 (3) always have a ratio of more than five, similar to positive control NB-4. M3 (2) shows the lowest positive/negative ratio of the M3 samples. Compared to the negative patient samples M4 and M5 the ratios of M3 (2) are higher. This is also shown in Figure 7 and Figure 8. The RFU values for the M3 (2) sample, both inside and outside the microfluidic chip, are higher than the values of the M4 and M5 samples.

The slightly increased fluorescence intensities of negative patients’ samples in comparison to negative cell lines could indicate a low concentration of the fusion protein PML-RARα. However, previous analyses exclude the presence of PML-RARα. Therefore, it is very likely that no fusion protein is present in the samples M4 (1) and M5 (1) and the increased fluorescence intensities are based on matrix effects of the samples. Matrix effects are the sum of all effects that influence the measurement of the analyte PML-RARα due to the different components in a sample. The patient samples were obtained from the patients’ bone marrow, which is not the case for negative cell lines. Due to different matrices, unwanted interactions of the assay antibodies may occur. This may be caused by different viscosities, nutritional habits of the patients or different proteins in the samples.

In summary, each of the patient samples declared positive have a higher fluorescence intensity than the patient samples declared negative and the negative cell lines. In order to define a threshold for declaring a patient sample as positive or negative, further analyses would have to be performed on a larger cohort of patient samples. However, using a small number of patient samples, we could prove the biological principle for a rapid testing of PML-RARα in a microfluidic system as a powerful diagnostic tool at an early stage.

Dekking et al. have shown, with the development of an immunobead assay for the translocation product PML-RARα on protein level, that this can be used for the diagnosis of APL [19]. The immunobead assay is performed in approximately 4 h and requires a flow cytometer for detection [19]. This assay is able to detect different forms of PML-RARα independent of break point position in the fusion gene [19]. The developed bead-based sandwich ELISA presented in this paper can be performed four times faster and requires only a multiwell plate reader for fluorescence detection.

The analysis for PML-RARα can be automated with the presented method. Only the sample preparation is done manually. The liquid handling system and the microfluidic channels make this possible. In summary, the process in the microfluidic chip offers the following advantages over the process in the reaction vessel. Washing steps no longer have to be performed manually. Complete process automation is possible after sample preparation. The diffusion rate and the surface-to-volume ratio are increased by the microstructure. This, in turn, increases the reaction speed and provides a better positive-to-negative signal ratio. The developed assay can be performed in less than 1 h. Miniaturization also means that less sample material and reagents are required. In the experiments described above, 98.5 % fewer assay components were used.

The microfluidic based method can also be transferred into a compact complete system and would allow a diagnosis independent of location. The elimination of manual work steps means that less demands are made on the qualification of staff. The bead-based sandwich ELISA could be used in future for the rapid diagnosis of APL at an early stage in order to quickly start a tailor-made indication therapy.

The developed assay scheme in combination with the microfluidics and the liquid handling system can be adapted to different analytes. A modular design of the system makes this possible. For the detection of another analyte, e.g., another biomarker for leukemias, the specific antibodies have to be replaced. This includes the capture antibody and detection antibody. The new antibody system must then be validated and checked for parameters such as specificity and sensitivity. Such a modular system consisting of many consistent elements, which can be used for the diagnosis of various leukemia-specific biomarkers, would have the advantage of considerably improving the diagnostic possibilities of medical practices, small hospitals and facilities in infrastructurally weak areas. Diagnostic tests would be more widely available, which would have a positive impact on the time needed for diagnostics and would improve overall medical care.

## 4. Materials and Methods

### 4.1. Materials

PML antibody (PG-M3) (Santa Cruz Biotechnology, Dallas, TX, USA), RARα antibody (C-1) (Santa Cruz Biotechnology, Dallas, TX, USA), Roti-MagBeads Streptavidin (Carl Roth, Karlsruhe, Germany), Low-Cross-Buffer (CANDOR Bioscience, Wangen im Allgäu, Germany), bovine serum albumin (BSA) (Carl Roth, Karlsruhe, Germany), Roti quant universal (Carl Roth, Karlsruhe, Germany), Biotin-NHS (Thermo Fisher, Waltham, MA, USA), PBS-Buffer (AppliChem, Darmstadt, Germany), Tween 20 (Bio-Rad, Hercules, USA), NB-4 (DSMZ, Braunschweig, Germany), HL-60 (DSMZ, Braunschweig, Germany), MV4-11 (DSMZ, Braunschweig, Germany), IP-Lysis-Buffer (Thermo Fisher, Waltham, USA), Protease inhibitor cocktail (Thermo Fisher, Waltham, MA, USA), FBS (Gibco Thermo Fisher, Waltham, MA, USA), Pen/Strep (Gibco Thermo Fisher, Waltham, MA, USA), RPMI Medium (Gibco Thermo Fisher, Waltham, MA, USA), QuantaRed Enhanced Chemifluorescent HRP Substrate (Thermo Fisher, Waltham, MA, USA), syringe pumps (World Precision Instruments, Sarasota County, FL, USA), syringes (B. Braun Melsungen, Melsungen, Germany), 3-way-valve (IDEX Health & Science, Oak Harbor, WA, USA), motor selection valve (IDEX Health & Science, Oak Harbor, WA, USA), tubes (Carl Roth, Karlsruhe, Germany), connectors (Carl Roth, Karlsruhe, Germany), multiwell plate reader infinite M1000Pro (Tecan, Männedorf, Switzerland), microscope axiovert 40 CFL (Carl Zeiss, Oberkochen, Germany), microfluidic chips (Micronit, Dortmund, Germany), chip holder for microfluidic chips (Micronit, Dortmund, Germany) multiwell plates (Thermo Fisher, Waltham, MA, USA), low-binding tubes (Bio-Rad, Hercules, CA, USA), Tecan i-control 1.9.17.0 (Tecan, Männedorf, Switzerland), LabVIEW 2018 (National Instruments, Austin, TX, USA), Microsoft Office (Microsoft, Redmond, WA, USA), magnet neodym 40 × 10 × 5 mm (Webcraft GmbH, Gottmadingen, Germany), pipettes 1–10 µL, 10–100 µL and 100–1000 µL (Eppendorf, Hamburg, Germany), Heraeus Megafuge universal centrifuge ((Thermo Fisher, Waltham, MA, USA).

### 4.2. Patient Samples and Ethics Statement

The study was reviewed and approved by the ethics committee of the medical association and the medical faculty of the University of Muenster (2007-524-f-S and 2007-390-f-S) before the study began. AML samples were obtained from bone marrow of patients with acute myeloid leukemia at the time of initial diagnosis. Informed written consent was obtained from all patients.

### 4.3. Cell Lines and Sample Preparation

Various leukemic cell lines were used for the development of PML-RARα detection in the microfluidic chip. The positive control was the cell line NB-4 with translocation t(15;17) validated by RT-PCR and for the negative controls we used the AML cell lines HL-60 and MV4 11. The cells were cultured under optimized conditions in RPMI medium with 10 % FCS and 1 % penicillin/streptomycin. For cell lysis, Thermo Fisher’s IP Lysis Buffer was used. The cells were washed 1x with PBS and resuspended in 500 µL IP-Lysis buffer per 50 µg wet cell pellet. The cell lysis was performed with 1 % protease inhibitors which were used to reduce protease activity and prevent degradation of the protein PML-RARα located in the cell nucleus. The lysates were incubated on ice for 5 min and then centrifuged at 4 °C and 13,000× *g* for 10 min. The AML samples were lysed in the same way as the cell line samples. The protein concentration was determined by the Roti-Quant universal assay and stored at −80 °C until further analysis.

### 4.4. Prototype Magnetic Bead-Based Sandwich ELISA

A sandwich procedure was chosen to increase the specificity of the assay. Both the anti-PML and anti-RARα antibodies had to bind to the fusion protein PML-RARα for a significant positive signal. The bead-based sandwich ELISA was performed only with one incubation step with all reagents except the HRP substrate. The incubation step was followed by washing steps and a detection step in which the HRP substrate was added. Roti-MagBeads coated with streptavidin were washed two times with PBS with 0.05%Tween 20 and then one time with PBS. The anti-PML-biotin antibody and the anti-RARα-HRP antibody were diluted in PBS with 0.5% BSA to a concentration of 1 µg/mL. For one diagnostic detection of the presence of PML-RARα, 5 µL Roti-MagsBeads with a concentration of 10 mg/mL were used. An amount of 25 µL of the anti-PML and anti-RARα antibodies with a concentration of 1 µg/mL were added to each reaction batch. Furthermore, 50 µL of low-cross-buffer and 100 µL of the cell lysate with a concentration of 100 µg/mL were added to each reaction. The reaction mixture was mixed well and incubated under rotation for 30 min at room temperature in a low-binding tube or a multiwell plate. After incubation, the beads were collected at the magnet, the supernatant discarded and at least three washing steps with PBS were performed. Afterwards 60 µL of the HRP substrate QuantaRed was added for two minutes to the washed beads. After two minutes, 50 µL of the supernatant was transferred to a microtiter plate to measure the fluorescence intensity. The fluorescence intensity was detected with a multifunctional monochromator-based microplate reader at excitation wavelength of 570 nm and an emission wavelength of 585 nm in a multiwell plate. The analysis of the patient samples outside the microfluidic chips was performed in a multiwell plate.

### 4.5. Transfer to the Microfluidic Chip

First, washing steps for magnetic beads in the microfluidic channel were established. The magnetic particles were injected into the channel. Then, the microfluidic was positioned on a magnet with its channels parallel to the horizontal magnet orientation. Wash solutions were pumped through the channel while the magnetic particles were held along the channel wall. After injection of the wash solution, the channel was moved vertically over the magnet, so that the beads were dispended in the channel. In the next process step, the beads were collected again on the channel wall, the buffer was removed and the next assay liquid could be added.

The magnetic particles were injected into the channel and washed three times. Then a mixed solution was injected consisting of 1 µL capture antibody (anti-PML-biotin) with a concentration of 1 µg/mL, 1 µL detection antibody (anti-RARα-HRP) with a concentration of 1 µg/mL, 2 µL low-cross-buffer and 4 µL cell lysate with a concentration of 100 µg/mL. This reaction mixture incubated for 30 min at room temperature with the washed Roti-MagBeads in the microfluidic channels. After at least three washing steps of the beads in the channel the substrate, QuantaRed was added and incubated for 2 min. Then the fluorescence intensity was detected. The detection in the microfluidic chip was also performed in the multifunctional microplate reader.

A special layout was designed for the measuring points along the microfluidic channels. For each channel, 20 measuring points were defined. For the 10 measuring points in the middle of a channel, a mean value was calculated as the result. This should have reduced the influence of the beads and the distribution of the substrate in the channels on the measurement.

### 4.6. Microfluidic Chip Design and Production

The microfluidic chips were designed and manufactured by Micronit GmbH (Dortmund, Germany). D263 glass wafers were used, which were microstructured by photolithographic methods and etching steps. The structured wafers were then thermally bonded with unstructured glass wafers. The inlets and outlets were created by sandblasting. The channels were 40 mm long, 500 µm wide and 100 µm deep. The inlets and outlets had a diameter of 500 µm. An individual chip holder was designed to fill the channels.

### 4.7. Liquid-Handling-System

The microfluidic chip, the chip holder, syringe pumps, valves, multiple tubes and connectors built the liquid-handling-system. This allowed us to fill each microfluidic channel on the chip with the liquids in the syringes of the pumps. Both the pumps and the motor selection valve were controlled by a LabView program and were optimally adapted to the process. An automated execution of the assay via the liquid handling system was thus possible. Washing steps were performed automatically under precisely controlled conditions by using the liquid handling system.

## Abbreviations

PML	promyelocytic leukemia
RARα	retinoic acid receptor α
RT-PCR	reverse transcriptase polymerase chain reaction
ELISA	enzyme-linked immunosorbent assay
µTAS	micro total analysis system
POC	point-of-care
APL	acute promyelocytic leukemia
AML	acute myeloid leukemia
FISH	fluorescence in situ hybridization
FAB	French–American–British classification system
M3	FAB classification of acute myeloid leukemias—M3 promyelocytic
ATRA	all-trans-retinoic acid
ATO	arsenic trioxide
HRP	horseradish peroxidase
RS232	recommended Standard 232
RFU	relative fluorescence unit
PBS	phosphate buffered saline
BSA	bovine serum albumin
